# Duplication mutation in CHIT1 gene is associated with poor response to medical therapy in patients affected with filarial chyluria

**DOI:** 10.6026/97320630016688

**Published:** 2020-09-30

**Authors:** Shriya Pant, Apul Goel, Pravin Kumar Gangwar, Prashant Gupta, Akancha Pandey, Satya Narayan Sankhwar

**Affiliations:** 1 Department of Urology, King George's Medical University, Lucknow, UP, India; 2 Department of Microbiology, King George's Medical University, Lucknow, UP, Inida; 3 Department of Obstetrics and Gynaecology, King George's Medical University, Lucknow, UP, India

**Keywords:** Lymphatic Filariasis, Chitotriosidase gene, Triglycerides, Infection, Genetic polymorphism

## Abstract

We explore the impact of CHIT1 gene mutation on clinical, biochemical parameters and response to outcome (remission/failure) of medical treatment in North Indian filarial chyluria (FC) patients. Data of 101 subjects of FC treated medically between March 2013 and April 2016 in whom CHIT1 gene polymorphism was determined were analyzed. Filarial etiology was confirmed by DEC-provocative test, immuno-chromatographic test and IgG/IgM-combo rapid antibody test. CHIT1 gene polymorphism was genotyped by polymerase chain reaction. Of 101 patients (mean age, 36.9±10.28 years; male: female, 3:1.2), 66 experienced remission (Group-A) while 35 experienced relapse or failed to respond (Group-B). A significant association was observed between CHIT1 genotypes and higher grade of disease (p= 0.001). Wild-type, heterozygous and homozygous mutant frequencies of CHIT1 genotypes were 78.6%, 72.5% and 27.8% in remission and 21.4%, 27.5% and 72.2%, in recurrence/failure, respectively. Our results showed that patients with mutant genotype (TT) of CHIT1 gene showed significantly higher rate of recurrence or failure to medical therapy than wild type (HH) genotypes [OR (95% CI) = 9.53 (1.84-49.21), p=0.011]. This preliminary study showed the impact of CHIT1 gene variants on treatment outcome in FC patients. This observation needs to be confirmed using studies with larger numbers of FC patients.

## Background

Chyluria is the presence of milky-white lymphatic fluid that is rich in protein and dietary lipids in the form of chylomicrons in urine. Chyluria is usually due to thoracic duct obstruction, which arises from a variety of causes, including filariasis, trauma, surgical procedure, tumour and aneurysm [1]. Chyluria occurs in 10% of filarial-affected patients. Filarial chyluria (FC) is frequently reported from Southeast Asia, China, India, Japan, Taiwan, Sub-Saharan Africa, South and Central America. About one third of infected people live in India. It has been recognized as a neglected tropical disease that is more prevalent in the rural and poverty-stricken population [2,3].

The natural history of FC is characterized by remissions and relapses. Sometimes, it resolves spontaneously with bed rest/or use of abdominal binders that increase intra-abdominal pressure to stop lymph leakage. Prolonged chyluria may result in loss of weight, lymphopenia, hypo-proteinemia, and anaemia. Medical treatment consists of high-protein and low-fat diet with medium-chain triglycerides (TGs), high amount of fluid intake and anti-filarial drugs [4]. Although the cause of FC is well-known but reason for susceptibility is unknown. In an effort to identify the reason for susceptibility for FC we reported an association between 24-base pair (bp) duplication mutation in exon10 of chitotriosidase gene (CHIT1) and susceptibility to FC [5]. This association may be due to variation in the coding region of CHIT1 with a 24-bp duplication allele reducing CHIT1 activity. This may enhance survival of filarial parasites. CHIT1 gene has been implicated as a predisposing factor for other infectious diseases also [6]. FC is characterized by unpredictable response to treatment with some patients responding well while others showing poor response. The reason for this is also unknown. Efforts have been made to find predictors of response to medical therapy by evaluating the various clinical and biochemical parameters [4].

In our endeavour to look for genetic predictors of response to medical therapy, we evaluated our data to see whether CHIT1 gene also plays a role in determining the natural history of FC and its response to treatment. The present study was conducted to find an association of CHIT1 genotypes with clinical presentation, biochemical parameters and response to treatments. To the best of our knowledge a link between genetic variation and treatment response in patients with FC is yet to be investigated. Knowledge about the natural history of this disease and the predictors of response will not only improve treatment decisions but also help in understanding the basic patho-physiology of this neglected disease.

## Materials and Methods:

### Study Design and Subjects:

This prospective study was conducted between March 2013 and April 2016 (including patient recruitment between March 2013 to April 2016 and follow-up till November 2017) at a hospital located in filariasis endemic zone. Ethical clearance was obtained from the Institutional Ethics Committee (Reference code number: 5534/R.cell-13). After obtaining written informed consent from patients, data was collected using a standardized questionnaire. Patients older than 18-years with FC, confirmed by either of the following tests, viz., specific diagnostic tests using Giemsa-stained thick and thin smear examination followed by DEC-provocative test, immuno-chromatographic card test (BinaxNOW® filariasis, Alere, North America, Orlando, USA) and IgG/IgM combo rapid antibody test (CTK Biotech Inc., CA, USA) were recruited [7-10].

Patients below 18-years age, non-chyluric whitish-cloudy urine, non-parasitic chyluria, malignancy, pregnancy, renal failure and uncontrolled diabetes were excluded [11]. Similarly, if evidence of parasitic infection could not be demonstrated by above listed tests, then chyluria was considered as non-parasitic and such individuals were excluded. Data recorded included history, details of previous episodes, treatment taken and response to treatment. Urinary investigations included routine urinalysis, culture, presence of chyle (gross assessment, ether dissolution test followed by microscopy), and quantitative analysis of urinary TGs and cholesterol levels using the biochemical auto-analyzer.

The severity of chyluria was graded according to prevalent clinical grading system incorporating slight modifications as per the severity of hematuria as mentioned below: [12-14]

[1] Grade-I: Single episode or ≤ 1-episode/year of hematuria of < 24 h duration, not associated with the passage of blood clots or anemia.

[2] Grade-II: Single episode or ≤ 1-episode/year of hematuria of ≥ 24 h duration, not associated with the passage of blood clots or anemia.

[3] Grade-III: ≥2 episodes/year or hematuria associated with the passage of blood clots or anemia.

Patients were initially managed with anti-filarial drugs, supportive medicines like hematinics (if needed), rest and dietary modifications. Anti-filarial drug regimen included 1-2 courses of diethylcarbamazine (DEC) in a dose of 6 mg/kg/day (three divided doses) for three weeks, doxycycline 100 mg twice a day for 3-weeks, single dose of albendazole and ivermectin with 15 days interval between the two courses. Prescribed dietary modifications included high-protein diet, green leafy vegetables, restricted fat intake (25-50 g/day) with the inclusion of fats containing medium-chain triglycerides and high fluid intake. A diet chart mentioning general guidelines for dietary modification was given to all patients. However, no specific recommendations regarding precise amounts or particular food items were given. After collecting the above details, 5 ml peripheral blood was obtained from each subject to perform diagnostic tests and molecular analysis as described previously [5].

### Genotyping:

Genomic DNA was extracted from whole blood using commercially available DNA isolation kit (Quick- g DNATM, USA) as per manufacturer's protocol. 100-ng of DNA was used as template for subsequent PCR reactions. Genotyping of CHIT1 gene was carried out using polymerase chain reaction with specific primer pairs as described in our previous study [5].

### Follow-up Protocol:

Patients were initially followed at 1-month followed by a 3-month visit. They were then advised to visit the clinic in case of relapse or problem. Those patients who could not attend the clinic for follow-up were enquired telephonically regarding recurrence. Successful treatment was defined as complete resolution of symptoms with stable remission until last follow-up. Patients not responding to prescribed regimen of two courses of drug combinations along with appropriate dietary and supportive measures or those presenting with recurrent symptoms after initial response were regarded as recurrence/failure. Genotype frequencies of FC patients were compared with demographic, clinical, biochemical parameters and outcomes (remission/recurrence) of medical therapy.

### Statistical analysis:

Analysis was performed using SPSS (Statistical Product and Service Solutions) version-17. Categorical data was summarized as number and percentage. Continuous data was expressed as mean ± standard deviation (SD). The chi-square test (χ2) and Kruskal- Wallis Test were used to assess association. Correlation of CHIT1 genotypes with response to medical therapy was analyzed by online web tool (http/www.vassarstats.net) by using Fisher exact probability test. P value less than 0.05 (p<0.05) was considered to be statistically significant.

## Results:

One hundred eight patients fulfilled the inclusion criteria. Seven patients were lost to follow-up immediately after treatment and were excluded from analysis. Thus data of only 101 patients was analysed. Basic characteristics of 101 FC patients are summarized in Table 1 (see PDF). These patients with mean age ± standard deviation [SD] 36.90 ±10.28 underwent medical therapy. Grade II (51.5%) chyluria was a most common presentation followed by Grade I (38.6%) and Grade III (9.9%). Urinary TGs and cholesterol (mean ± SD) were 229.28± 181.82 and 19.28± 17.46 g/dl, respectively. Of the 101 patients, 66 (61.11%) experienced and continued to be in remission till last follow-up (Group-A). 9 patients failed to respond to medical treatment and were labelled as treatment failures while 26 patients experienced recurrence of symptoms after a initial response. These two categories of patients were clubbed in to Group-B. Therefore, overall success rate for medical therapy was 65.35%.

The association of CHIT1 genotypes (wild: HH /heterozygous: HT/ mutant: TT) with clinical and biochemical data is summarized in Table 2 (see PDF). Urinary TGs level was insignificantly higher in HT/TT genotypes when compared with HH genotype. Moreover, patients with HT/TT genotypes had higher grade of chyluria (grade II/III) and this difference was statistically significant (p=0.001). The association of CHIT1genotype with response to medical therapy was analysed and summarized in Table 3 (see PDF). The wild type, heterozygous and mutant genotype frequencies were 78.6%, 72.5% and 27.8% in remission (Group A) and 21.4%, 27.5% and 72.2% in recurrence/ failure (Group B) patients, respectively. Our results showed that the patients with mutant genotype had a higher risk of recurrence or failure to medical therapy [OR (95% CI) = 9.53 (1.84-49.21), p=0.011].

## Discussion:

Chyluria is a urological manifestation of lymphatic system abnormality. This tropical, debilitating disease is known for recurrence. Lymphatic filariasis is the world's second leading cause of long-term disability [15]. The current estimate reveals that 120 million people in 83 countries of the world are infected with lymphatic filarial parasites. About 23 million people suffer from filarial disease in India with high endemicity in our province, Uttar Pradesh (14.6%). Approximately 10% of filariasis patients develop chyluria About 30%-40% of patients do not respond or respond partially to medical therapy or recur after the initial response [3,4]. Most recurrences are treated by repeat course of conservative medical treatment [16]. However, some of these patients need endoscopic sclerotherapy.

Although not uncommon, the disease has escaped the attention of investigators with few molecular or genetic studies reported. The polymorphisms of host defence pathway genes, including MBL, VEGF, HLA, TLR, PDL-1i and IL-10RB are associated with susceptibility and clinical status of the disease [17-20]. Chitin has an important role for embryogenesis in adult worms and is a component of microfilaria sheath. Human chitotriosidase (CHIT1) is a chitin-degrading enzyme, which provides a protective role against chitin-containing pathogens. Data suggest that polymorphisms of CHIT1, contributing inactive CHIT protein, might increase susceptibility to lymphatic filarial parasites [5]. CHIT1 deficiency, due to the polymorphisms, increases the susceptibility to bancroftian filariasis in southern Indian population. Our hospital is located in filarial endemic zone and urologists are primarily involved in treatment of patients with chyluria. Although the whole population within endemic regions is exposed to the parasite, clinical disease only develops in certain individuals. To answer this question, our group studied the CHIT1 and MBL2 gene polymorphisms in patients of FC and age- and sex-matched controls living in the same geographical region. In our previous study we found the significant association of CHIT1 polymorphisms with susceptibility to FC. Carrying the same study forward we analyzed our data to see if these genetic polymorphisms also predict the severity and clinical response to treatment.

Evaluating the efficacy of treatment is complicated by the unpredictable duration of spontaneous remission. Also, recurrences are known to occur after many years. All the patients are counseled about the need to avoid mosquito bite. However, this precaution is difficult to follow for patients of low socio-economic strata. Additionally, ongoing mosquito bites may change the natural history of this condition. Another problem in studying this patient group is the loss to follow-up as this disease is not fatal and is painless. Our group has been trying to explore the clinical predictors of recurrence of FC knowing fully well the pitfalls of this data [8]. In our present study we explored the same endpoint and recruited a different population of patients. Additionally, we evaluated the impact of genetic alterations that might influence the risk of recurrence. This aspect of our study is unique and has never been reported before.

In this study we looked at the genetic loci to unravel the predictors of response to medical treatment. On analyzing the association of CHIT1 genotypes [wild (HH), heterozygous (HT) and mutant (TT)] with clinical, biochemical and treatment response data, no differences were observed in total disease duration and number of previous attacks. However, a significant association was observed between CHIT1 genotypes and higher grade of disease (p= 0.001). Of the 101 patients, wild type, heterozygous and mutant genotype frequencies were 78.6%, 72.5% and 27.8% in remission (Group A) versus 21.4%, 27.5% and 72.2% in failure (Group B) patients, respectively. 78.6% patients with remission had wild genotype (HH) while 72.2% patients with failure had mutant genotype (TT). In this study, CHIT1 gene mutant genotype (TT) showed significant association with recurrence/failure to medical therapy [OR (95% CI) = 9.53 (1.84-49.21), p=0.011]. Our results show patients with a mutant genotype had a greater risk of recurrence or of failed medical intervention.

Our study has some limitations like small sample size and short follow-up. Out of the different manifestations of LF we chose only FC. This was done because urologists (study was conducted in Department of Urology) primarily manage patients of chyluria. There are limitations in the serological tests used for the diagnosis of filarial etiology. Also, the unpredictable nature of this disease makes interpretation of data difficult Multivariate analysis was not performed, as the number of samples in this study is low, with the risk of generating false positive results. However, our study is a preliminary study that has looked into a unique aspect of this condition. There is a need for more studies with a larger pool of single nucleotide polymorphisms, which could unravel the genetic basis of response to medical therapy.

## Conclusion:

We found that homozygous wild genotype (HH) of CHIT1 gene was associated with improved response while mutant genotype (TT) was associated with poor response to medical therapy. This observation supports the relationship between CHIT1 genotypes and treatment outcome. More studies in multiple cohorts are required to confirm the importance of CHIT1 gene polymorphism on the response to treatment.
